# Supplementary figures and images for: Reexposure to a sensorimotor perturbation produces opposite effects on explicit and implicit learning processes
Source: PLoS Biol. 2021 Mar 5;19(3):e3001147. doi: 10.1371/journal.pbio.3001147 (PMC7968744; doi:10.1371/journal.pbio.3001147)

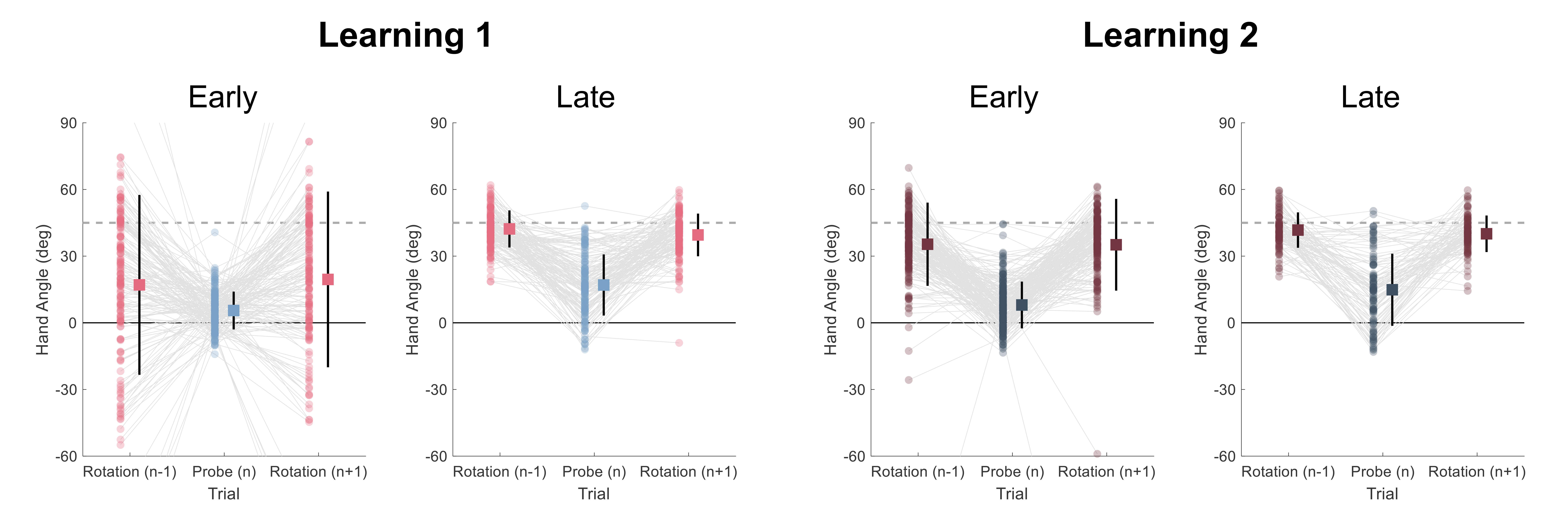

Supplement: S1 Fig — Hand angle for each trial in Rotation–Probe–Rotation triplets, highlighting the switching behavior for each participant (gray lines) between the Rotation (pink dots) and Probe trials (blue dots). The horizontal dashed gray line represents full compensation for 45° visuomotor rotation. The data are shown for early and late stages of the 2 learning blocks. The mean values (squares) increase from early to late, and within the early phase, between the first and second block on Rotation trials, indicative of overall learning and savings, respectively. Black vertical lines indicate standard deviations. The individual data and the summary statistics presented in this figure can be found in S1 Data. The raw data can be found in https://git.io/Jtiip. (TIF) [file pbio.3001147.s001.tif]
