# Supplementary material for: Reexposure to a sensorimotor perturbation produces opposite effects on explicit and implicit learning processes
Source: PLoS Biol. 2021 Mar 5;19(3):e3001147. doi: 10.1371/journal.pbio.3001147 (PMC7968744; doi:10.1371/journal.pbio.3001147)
Supplement: Attachment — Submitted filename: Avraham_ResponseToReviewers.docx [file pbio.3001147.s005.docx]

RE: Manuscript Number PBIOLOGY-D-20-02151R2

**Editor:**

Your revisions should address the specific points made by each reviewer. As you will see from the reviewers’ comments, all of them feel that the suggestion that implicit adaptation during relearning demonstrates attenuation is both interesting and thought-provoking. However, the reviewers also raise a variety of concerns, particularly with your Experiment 1, that lead them to question the strength of this conclusion. Reviewer 1 challenges the evidence for implicit adaptation, and the reviewers together ask that you provide additional support for your conclusions with additional clarifications and analyses, as well as new task designs and experimental data. Reviewer 2 also asks that you provide clarification as to whether the attenuation of implicit savings is a context-dependent/task-specific effect or is a more general phenomenon. While this reviewer indicates they’d like you to discuss this, for the broad readership of PLOS Biology, we feel that this concern would also be better assessed with additional data. We think that if you provide new and better evidence, you might be also addressing reviewer 3’s criticism.

We thank the editor and the reviewers for their constructive comments. We have revised our manuscript in response to these comments and suggestions.

To address the major concerns of the reviewers, the revision includes the following additional experimental data:

- We performed a new experiment of similar design to Experiment 2, using the clamped-feedback manipulation. Given constraints imposed by the pandemic, we conducted this experiment online using a crowdsourcing platform. We had concerns about moving sensorimotor adaptation work online given the variation in stimulus presentation and response devices (e.g., mouse or trackpad), as well as absence of a good method to ensure that the participants pay attention. However, we piloted the platform on a range of tasks and then ran the study using a design similar to Experiment 2. The results are very similar to those obtained in the in-lab study, showing attenuation of implicit adaptation upon relearning during the learning and aftereffect blocks.
- We expanded our review of the literature, doing our best to consider every study that we thought provided an appropriate test to look at savings/attenuation under implicit conditions. We are hesitant to call this a meta-analysis given that the number of studies meeting the criteria is limited (9 papers, 13 experiments). Nonetheless, we present the results of all of these experiments and, overall, find a consistent pattern of attenuation upon relearning.

Below is a point-by-point response to the comments. Throughout the text, we use the following color-coding:

- Original comments by the reviewers- black regular font
- Our responses- blue regular font
- **Citation from the paper- “blue bold font in quotation marks”**
- *Figure captions- blue italic font*

Line numbers refer to the revised version of the paper.

We have also attached a redlined document that highlights the changes in the manuscript from the original submission.

Thank you for your consideration.

**Reviewer #1:**

The authors report an interesting phenomenon in which implicit adaptation to a visuomotor rotation becomes attenuated when participants relearn the rotation after the previous adaptation is washed-out. This phenomenon stands in contrast to explicit adaptation, in which the learning becomes faster during the relearning (known as "saving"). Their experiments were carefully designed, and the data support the authors' conclusion. The manuscript is well-written. Although the mechanisms underlying the above attenuation are hypothetical ones at this stage, the findings would be provocative in the field of sensorimotor learning. Thus, this study seems to be appropriate for "Short Report" in this journal. However, there are several issues to be clarified as follows.

Major concerns:

1. Clamp trial as a measure of implicit adaptation. One of the critical assumptions in this study is that the performances of participants in clamp trials are measures of implicit adaptation. The authors referred to previous studies supporting this assumption. However, they need to clarify the validity of this assumption in this manuscript.

We thank the reviewer for pointing out that we should not assume the reader is familiar with the clamp method nor arguments for why this method is assumed to isolate implicit adaptation. We have expanded our discussion of the method and also include some additional analyses relevant to the issue of whether behavioral changes observed in this task are implicit.

An integral part of the task, other than the invariant nature of the feedback, are the instructions, emphasizing that the participants should always aim to reach directly to the target and ignore the cursor feedback. While we did not probe aiming on every trial, we did include a post-experiment survey, where we asked “Did you ever try to reach to places other than the target?”. None of the participants reported that they consistently used an aiming strategy other than to reach to the target.

To evaluate awareness of the change in behavior, we also asked “Do you think you changed your behavior at all during the experiment?”. We purposely use a general (and vague) question to avoid biasing the participants’ answers; thus, the responses can be quite variable and idiosyncratic. However, they do not suggest awareness of adaptation: 44% of the participants answered ‘no’. The majority of the others commented that they thought they became better at the task, reaching faster and more accurately, and most of the other comments were about becoming fatigued. One participant described “started flailing more” because he/she did not have control over the cursor. None of the reports discussed a systematic change in hand direction.

In addition to summarizing the survey results, we have also added an analysis that supports the assumption that the behavioral changes to the clamped feedback are implicit. The aftereffect (measured on no-feedback or veridical feedback trials) is generally assumed to provide the best probe of implicit adaptation since it captures an unintended bias in behavior as participants aim for the target (Mazzoni and Krakauer, 2006; Taylor and Ivry, 2011, 2014). We looked at the relationship between asymptotic performance (mean hand angle over last 10 cycles of the clamp block) and the aftereffect (hand angle during the first cycle of the no feedback block), calculating the ratio between the two measures for each participant. A value of 1 would mean perfect correspondence, although we would expect the value to be slightly less than 1 if all learning is implicit but there was some forgetting during the first aftereffect cycle (Zhou et al., 2017; Hadjiosif and Smith, 2013, Hadjiosif et al. 2014). The results (means and 95% confidence interval) were in line with this prediction: Test Group- 1^st^ learning block: 0.94, [0.88 1.00], 2^nd^ learning block: 0.85, [0.74 0.96]; Control group: 0.95, [0.82 1.08].

Further evidence supporting the assumption that participants aimed to the target throughout the experiment comes from the reaction time (RT) data. Reaction times in visuomotor rotation tasks increase with changes in aiming strategy (McDougle and Taylor 2019), and this is evident in the RT data from the rotation trials in experiment 1. With the clamp manipulation in experiment 2, the reaction times were uniformly fast (~400 ms) and comparable across the different phases of the experiments (Clamp vs Aftereffect: t(15)=0.810, p=0.430, BF_10_=0.340, d=0.20; Learning 1 vs Learning 2: t(15)=-0.390, p=0.702, BF_10_=0.273, d=-0.10).

We now clarify these points in the paper, attempting to be concise in summarizing this information (lines 203-221 and 686-689).

2. Rationales for parameter settings. It seems that the authors carefully selected parameters in the two experiments based on their long-standing experiences on the visuomotor rotations. It was not clear why they set the rotational angle to 45º in Experiment 1, or why they selected 15º as the fixed angle of the clamped feedback.

We chose to use a 45° rotation in Exp 1 because a perturbation of this size has been shown in various studies to 1) reliably produce savings (e.g., Morehead et al., 2015; Zarahn et al., 2008), and 2) entail behavioral changes that involve a substantial contribution from explicit and implicit learning processes (e.g., Bond and Taylor 2015). The case is more ambiguous for smaller perturbations such as 30° (e.g., Krakauer et al. 2005, Hinder et al. 2007, Kitago et al. 2013, Morehead et al. 2015). Thus, we opted to use a 45° perturbation given our desire to be able to estimate “savings” for both processes.

A clamp size of 15° was somewhat arbitrary. Prior studies (Morehead et al. 2017, Kim et al. 2018) with the clamp method suggests that implicit adaptation is invariant for clamp sizes above 10° (or even a bit lower), and this invariance is further supported by studies using a random, contingent feedback (based on the trial-by-trial response, Wei and Kording 2009). Given this literature, we expect we would obtain the same results if we had used a larger clamp.

We added this information to the paper (lines 591-593, 646-648).

3. An interaction effect between block and cycle. The authors stated that change in hand angle was larger early on in the second learning block, but this advantage was transient and eventually reversed in the probe trials of Experiment 1 (Lines 171 - 172). However, they need to confirm a significant interaction between block (learning or relearning) and cycle before this statement.

In designing the study, we planned to conduct a two-part analysis. The first was motivated to follow conventions in the savings literature, using pre-defined windows for early and late adaptation. The second was the cluster-based analysis, a statistical method we have been advocating for learning studies in our recent papers (e.g., Labruna et al. 2019, Tsay et al. 2020). For the pre-defined window, our plan was to use t-tests for the early and late windows. In response to the reviewer’s comment, we now also report the results of a 2 (Early/Late) x 2 (First/Second block) for each experiment along with the t-tests. Of relevance to the reviewer’s question, the interaction was significant in Experiment 1. However, while the changes in hand angle between the blocks exhibit a numerical reversal, the simple effect t-tests showed a significant effect for Early and only a marginal effect for Late. As such, we softened the claim about a reversal. We edited the text as follows (lines 129-138):

**“In the pre-defined stage analysis, hand angle increased within each block, indicative of the operation of implicit adaptation (Stage main effect: F(1,23)=45.0, p<0.001, η_p_^2^=0.66). However, the effect of block was not significant (F(1,23)=0.07, p=0.800, η_p_^2^=0.00). This null result is consistent with the proposition that implicit adaptation does not contribute to savings (see Morehead et al., 2015). This conclusion, however, is qualified by a significant interaction (F(1,23)=8.30, p=0.008, η_p_^2^=0.27): The change in hand angle was slightly faster in the early stage of the second learning block (2.79°±1.30°, t(23)=2.15, p=0.042, BF_10_=1.49, d=0.44, Fig. 1G), but marginally smaller for the late stage (-2.34°±1.20°, t(23)=-1.96, p=0.063, BF_10_=1.09, d=-0.40, Fig. 1H). In the cluster analysis, no significant difference was found between the learning blocks.”**

We also want to note here that, motivated by a comment from Reviewer #3, we modified the criterion used in the cluster analysis, opting to use a more conservative version of that test (see response to Reviewer #3 for details). Moreover, in implementing this, we realized that the first probe trial in the initial learning block occurs before the first rotation trial. As such, it is really a baseline trial and should not have been included in the analysis of the probe trials, an error we made in the initial submission and have now corrected. With the new criterion, the small clusters were no longer significant in the Probe trials of Experiment 1, but we see robust clusters showing attenuation in the aftereffect block of both Experiments 1 and 2, as well as in multiple cycles throughout the learning block in Experiment 2.

Finally, we note for the reviewer that we have added a supplemental experiment reporting an online replication of Experiment 2 and expanded our review of the literature. This review shows a pattern of attenuation of implicit adaptation (Fig. 3). Taken together, we believe the data presented in the revised manuscript make a compelling case that implicit adaptation is attenuated upon relearning.

Minor points:

Line 157: "a point that that would be farther …" This sentence was not understandable.

We have revised this section (lines 182-194).

Line 539 - 540: "… the second learning block, These probes …" Should the comma be replaced by a period?

We have made this correction.

**Reviewer #2:**

The current study investigated the effect of explicit and implicit components on savings, the phenomenon consistently reported in sensorimotor re-learning. With a series of elaborated visuomotor adaptation experiments to rotated cursor feedback, the authors have found that the implicit adaptation has the attenuated effect on the savings, which contradicts the previous reports.

By using a novel experimental method, the authors provide essential findings related to mechanisms about savings of visuomotor adaptation. The manuscript is well written, and the methodological approach and results are well explained. I have just two substantive points to address in revision:

Major comments:

1. The authors found that the implicit component is attenuated upon re-learning. Their finding is somehow surprising because this is incongruent with the recent papers which reported the implicit adaptation has a positive effect on saving. The authors have already acknowledged this fact by citing these studies and mentioned this discrepancy in the Discussion section. I wonder if the observed finding of attenuated implicit saving is context-dependent and specific to their current experimental setups, being not generally applicable to sensorimotor learning.

We thank the reviewer for raising this concern. We certainly did not expect to observe attenuation of implicit adaptation when we initiated this project. That said, we are confident that the attenuation is not limited to our experimental setups, but rather reflects a general phenomenon for visuomotor adaptation. First, Experiments 1 and 2 show attenuation despite the substantial differences in task designs (the use/non-use of explicit strategies, the presence of task performance error, overall changes in reach angles).

Second, we have now obtained a replication of Experiment 2. With the onset of the pandemic, our lab created an online platform for running motor adaptation studies, and based on a number of tests, the data from this approach is very similar to that obtained from in-lab studies despite the variation in experimental setups and response devices that are inherent to online research (Tsay et al., in prep). Subsequent to the reviews, we used the platform to conduct an online experiment using the clamped feedback manipulation of Experiment 2 (Experiment 2S). The results of this experiment essentially replicate those obtained in Experiment 2 showing attenuation of implicit adaptation upon relearning. This is now reported as a supplemental experiment with the data shown in Figure S2 (shown here).

***Figure S2. Experiment 2S: Replication study using an online crowdsourcing platform.***

*(****A****) Time course of participants’ (N=48) mean hand angle averaged over cycles, showing robust adaptation to the task-irrelevant clamped feedback (onset marked by the vertical lines) in blocks 1 (light green) and 2 (dark green) (Early-Late Stage main effect: F(1,47)=90.8, p<0.001, η_p_^2^=0.66). (****B****) Overlaid hand angle functions for the two blocks, showing attenuation in multiple clusters throughout the learning and aftereffect blocks (p<0.05). While the effect of Block was not significant (F(1,47)=1.09, p=0.302, η_p_^2^=0.02), there was a significant Stage x Block interaction effect (F(1,47)=4.99, p=0.030, η_p_^2^=0.10). (****C-D****) The interaction reflects the finding that there was a significant attenuation in the Late stage (-1.57°±0.74°, t(47)=-2.14, p=0.038, BF10=1.25, d=-0.31), but no difference in the Early stage (0.58°±0.61°, t(47)=0.950, p=0.347, BF10=0.240, d=0.14). (****E****) Similar to the results of Experiments 1 and 2, marked attenuation was observed in the Aftereffect stage (-3.35°±0.81°, t(47)=-4.14, p<0.001, BF10=168, d=-0.60). For panels C-E, the left pair of bars show the mean across participants for each block, and the right bar shows the mean of the within-participant differences Block 2 – Block 1. Black dots indicate individual difference scores. For all figure panels, shaded margins and black vertical lines represent standard error of the mean (SEM).*

Third, the literature review we performed and have now expanded entails a broad range of study designs and experimental setups. In expanding this review (a tack inspired by the comments of Reviewer 3, see below), we have attempted to include all studies we could find that were relevant to the question of how implicit adaptation changes upon relearning, and amenable to either re-analysis (by obtaining the original data from the authors, or from the published data) to determine the effect size. The review reveals a rather consistent pattern of attenuation, although for the most part this effect was either overlooked or only mentioned briefly in the publications (new Fig. 3, shown below). We even found evidence of attenuation within studies that superficially appeared at odds with attenuation, most notably the results from Yin and Wei (2020), who were kind enough to share their data with us (see Fig. 3B):

***Figure 3. Prior evidence for attenuation upon relearning for implicit visuomotor adaptation.***

*(****A****) Cohen’s d effect size of the differences in learning measures between the second and first learning blocks for experiments meeting our criterion for inclusion in the review of the literature. Early learning (green circles), Late learning (blue triangles), Aftereffect (purple squares) and Learning rate (dark purple stars). (****B, C****) Overlaid hand angle functions of implicit adaptation to a visuomotor rotation over two learning blocks separated by a washout phase (not shown). Light and dark colors denote blocks 1 and 2 of the experiment, respectively. Shaded margins represent the SEM. In B, implicit adaptation was extracted by subtracting a reported aiming location from movement hand angle on every trial. Adapted with permission from Yin and Wei, 2020. In C, implicit adaptive response to the rotated cursor when the target jumps in a manner that eliminates task error. Adapted with permission from Leow et al., 2020. (****D****) Learning rate during adaptation to a gradually changing visuomotor rotation, tested on two days. Pink markers represent individual participants. Black diagonal dotted line represents the unity line. Bars and black vertical lines (inset) represent mean and SEM, respectively. Adapted with permission from Stark-Inbar et al., 2016.*

We do acknowledge that, at present, we cannot evaluate if the attenuation phenomenon would generalize to other forms of sensorimotor adaptation. Currently, the tools for isolating implicit adaptation are better developed for visuomotor adaptation than for other adaptation tasks (e.g., force field, split-belt). Progress is being made in these other task domains, and for the most part, the results point to similarities across task domains; for example, recent work has shown that learning effects when reaching in force fields or walking on split-belt treadmills are also comprised of both explicit and implicit components (Hwang et al., 2006; McDougle et al., 2015; Schween et al., 2020; Roemmich et al., 2016). We think it is likely that attenuation of implicit adaptation may be found for these tasks, but that is, of course, an empirical question, one that we expect our paper will inspire researchers to take up.

The authors should discuss their current findings more in detail by explaining the specific mechanisms observed in their study.

We agree that explicating the mechanisms underlying attenuation is an important problem. The current study is focused on demonstrating this unexpected phenomenon and showing that it is robust, even if overlooked for many years. At present, we do not make strong claims to understand the mechanism, but we have sketched out a number of possibilities. These are addressed in the Discussion section, ‘*Possible mechanisms for attenuation upon relearning’* where we focus on describing two distinct hypotheses (lines 417-519):

1. Desensitization to the error signal. By this view, the sensorimotor memory is changed less by a previously experienced error.
2. Interference due to the interplay between two competing memories, one activated during recall when a perturbation is re-introduced, and the other linked to recent experience with an error of the opposite sign that was encountered during the washout phase.

In addition to describing these hypotheses, we also review research from other cerebellar-dependent learning phenomena that will be useful to consider in considering mechanisms.

2. One possible concern throughout the experiments is whether the participants accurately followed the given instructions. For example, in experiment 1, the authors used the two target colors (red or blue) to instruct the participants to explicitly use the different strategies. Although the authors emphasized the instructions by displaying the message during the task (experiment 1) or performing the demonstration of the clamped feedback trials (experiment 2), I wonder if these instructions are really followed by the participants. In addition, the instruction, target "will act differently", provides ambiguous information, and how the participants understood this instruction would affect their strategy. These concerns were not entirely addressed in this manuscript.

We agree that the conclusions we reach regarding attenuation of implicit learning upon relearning in both Experiments 1 and 2 (and the new Experiment 2S) assume that the participants followed the instructions. We believe that the data, as well as participants’ responses to a post-experiment survey, are consistent with this assumption.

In Experiment 1, the participants were instructed to move the **cursor** to the target when the target was red (rotation trials) and to move their **hand** to the target when the target was blue (probe trials). In line with previous studies (Morehead et al., 2015, Vandevoorde and Orban de Xivry, 2019), we deliberately did not provide participants with a specific reaching strategy on rotation trials. By remaining vague in our instructions, we assumed explicit aiming would develop in a manner similar to that taking place in a conventional visuomotor adaptation task, and we could then measure the dynamics of explicit and implicit contributions to behavior. The reliable difference in hand angle between rotation and probe trials (Fig. 1B) indicates that the participants changed their aiming direction and attempted to switch off strategy use on the probe trials. To highlight this switching between trial types, we show below the hand angles for individual trials during rotation-probe-rotation trial triplets (in the primary text we only show cycles of probe trials) from the early and late learning stage of both learning blocks. As can be seen, the plot shows striking changes in hand angle in response to the color cues.

***Figure S1. Experiment 1: Strategy switching between Rotation and Probe trials***

*Hand angle for each trial in Rotation-Probe-Rotation triplets, highlighting the switching behavior for each participant (grey lines) between the Rotation (pink dots) and Probe trials (blue dots). The horizontal dashed grey line represents full compensation for 45° visuomotor rotation. The data are shown for early and late stages of the two learning blocks. The mean values (squares) increase from early to late, and within the early phase, between the first and second block on Rotation trials, indicative of overall learning and savings, respectively.*

We have clarified our discussion on this issue in the revision when first describing the behavioral results during rotation and probe trials, as well as the statistical analysis for the comparison between these trials (lines 96-104). We have also added the above figure as a supplemental figure (Fig. S1). We opted to make this supplemental since the basic finding is conveyed by the data shown in Figure 1B.

However, we recognize that the design of experiment 1 does not provide the cleanest assessment of the implicit process **during the learning blocks**. This is because it required participants to frequently change strategy, and any failure to completely dispense with the aiming strategy would contaminate the probe trials. Such “switching errors” are widely recognized as unavoidable (Rogers and Monsell, 1995). These concerns were a primary motivation for conducting Experiment 2 since we believe the clamped-feedback method eliminates strategic re-aiming entirely, and thus provides a cleaner probe of implicit adaptation. We address these issues in the Results section (lines 155-167 and 195-197) and Discussion section (lines 395-404).

We have addressed the issue of compliance to instructions to clamped feedback (Experiment 2) in our response to Reviewer 1. In brief, the answers to the post-experiment surveys indicate that the participants aimed to the target, and importantly, did not mention use of an alternative aiming strategy. In addition, there is a tight linkage between asymptotic performance in the clamp block and the aftereffect, indicating that the estimates of implicit adaptation from clamped feedback obtained during learning are consistent with that obtained in the aftereffect phase. There is no reason to think that aiming would be relevant during the aftereffect phase. Lastly, consistent with the hypothesis that participants aimed directly to the target throughout the experiment, reaction times were uniformly fast (~400 ms) and comparable across different phases of the experiment. We added this information to the manuscript (lines 203-221 and 686-689).

Minor comments:

3. Please describe how the sample size was determined for each experiment.

In general, the sample size for each experiment was set to include ~30% more participants than the minimum required to ensure good statistical power and appropriate counter-balancing of the perturbation direction and target location sets. We now describe the process used to determine the number of participants in the protocol for each experiment (see details in lines 530-533, 570-575, 625-630 and 797-700):

4. I wonder if there is any difference between clockwise versus counterclockwise perturbations.

We have been keeping an eye on this question in many of the studies conducted in our lab. For the Rotation trials in Experiment 1, we did not find any difference in the degree of savings between the two rotation directions during early (t(22)=1.54, p=0.138) and late (t(22)=-0.24, p=0.812) learning. There was also no directional difference during early (t(22)=-0.96, p=0.346) and late (t(22)=-1.58, p=0.128) stages of the Probe trials, as well as no difference in attenuation of the aftereffect (t(22)=-0.32, p=0.752).

For Experiments 2 and 2S (online), we did not find clear differences in attenuation effects for clockwise and counterclockwise clamps. There was one place where the analysis of direction did yield a significant effect (we are using a loose statistical criterion here, not correcting for multiple comparisons): Attenuation was stronger for counterclockwise clamp during early learning in Experiment 2 (t(14)=-3.19, p=0.007). However, this effect was not observed in Experiment 2S (t(46)=-1.05, p=0.299). As such, we do not consider it reliable, and in fact, it does not survive a Bonferroni correction for the multiple comparisons entailed in these t-tests. In addition, attenuation observed during late learning and in the aftereffect phase was comparable between the two rotation directions in both Experiment 2 (late learning: t(14)=-0.38, p=0.711; aftereffect: t(14)=-0.27, p=0.794) and Experiment 2S (late learning: t(46)=1.26, p=0.213; aftereffect: t(46)=1.80, p=0.078).

We have summarized these null effects in the revision (lines 717-720).

5. It is not clear how the authors determined the number of trials for the extended washout block.

Our main goal in this paper was to examine whether implicit adaptation contributes to savings, meaning, to compare the two phases of learning. As such, it was important that we achieve full washout before the introduction of the second learning block. Previous work with contingent feedback (i.e., standard visuomotor rotation tasks) has shown that too short of a washout can create interpretation problems (Smith et al. 2006, Zarahn et al. 2008), and suggests using a washout block of at least the same length as that of the learning block (Morehead et al. 2015). Therefore, for Experiment 1, where the learning blocks consisted of 40 cycles, we opted to use 40 cycles of veridical feedback washout, along with additional 10 cycles for no feedback aftereffect block and multiple enforced breaks. We have expanded our description in the revision (lines 618-620).

For Experiment 2, we chose to use 100 cycles in the first learning block, assuming this would allow participants to reach asymptotic performance. Following a (short) no-feedback aftereffect block and a 2-min break, we employed a manipulation to produce washout in an implicit manner, one that would preclude awareness of adaptation. To this end, we used a clamp in the opposite direction, reminding participants to ignore the feedback. This readily drove behavior back to baseline. We opted to adjust the length of this reversed clamp phase based on the participants’ behavior, stopping the reversed clamp when the reaches were terminating close to the target. To ensure complete washout, we then used a veridical feedback phase (+ three 1-min breaks) with the net effect being 70 washout cycles (reversed clamp plus veridical feedback).

Importantly, as now emphasized in the paper, **“all of the participants in the Test group experienced at least 30 cycles (37.3±1.62) of veridical feedback before the second clamp block, ensuring they had sufficient exposure to unperturbed feedback prior to the onset of the second learning block.”** (lines 666-669).

The experimental protocol of the new Experiment 2S was based on that of Experiment 2. Based on pilot work, we opted to use 80 cycles for each learning block and, given our target experiment duration, this allowed us to include 90 cycles for the washout block (including the no feedback aftereffect block). This information is described in the paper (lines 701-704).

6. The last part of the Result section (page14, line 250-; "This attenuation of implicit learning, …") should better be moved to the Discussion part.

We thank the reviewer for this suggestion. We agree that this part was more appropriate in the Discussion section and have reorganized things in this manner. As mentioned above, this part was also edited and extended to include a broader review of the relevant literature (lines 307-415).

**Reviewer #3:**

This well-written paper shows the results of two experiments and describes the results from three previous studies that point to the attenuation in the implicit component of visuomotor adaptation upon relearning. The study is new and provoking because the existence of savings in visuomotor adaptation is commonly accepted, at least in abrupt perturbation conditions. The authors thus propose that only the explicit component exhibits savings. The results of experiments 2 are rather convincing, although the effect sizes (not provided) appear small. I do have however important reservations with the strength of the effect, notably in experiment 1.

Major

Both the design and the results of experiment 1 are relatively weak. About the design, I agree with the authors that the "failure to completely dispense with the aiming strategy would contaminate the probe trials" (line 180). This design thus creates a problem in the pure assessment of the implicit component. About the results, I do not agree that they "provide clear evidence of savings".  The reduced savings are indirectly inferred by the small between-group differences in the no-feedback block (figure 1I suggests a large variance, thereby a small effect size). In fact, the results show a small but significant increase, not decrease, in savings at the beginning of the relearning block.

We thank the reviewer for raising these concerns. To quantify the strength of the observed effects, we have added effect size information (Cohen’s *d* and η_p_^2^) for all of the statistics. We do want to point out that since we ask about savings, almost all of our analyses are for within-participant changes in behavior between the first and second learning blocks, not between-group effects. Also, our statement that Experiment 1 provides “clear evidence of savings” was intended to be in reference to the rotation trials only. We do believe the evidence of savings on the early rotation trials is clear (d=1.05, and a replication of earlier results). We have updated the text to clarify this point (lines 119-128, 156-158).

More important for the current paper is the robustness of the attenuation effect we observe for implicit adaptation, the core finding in this paper. Keeping in mind that the rule of thumb for Cohen’s *d* is 0.2=small; 0.5=moderate, 0.8=large, most of the comparisons fall in the moderate to large range when we look at the late learning and aftereffect data. In Experiment 1, the effect size is 0.40 for late learning and 0.70 for the aftereffect comparison. The late learning and aftereffect Cohen’s *d* values for Experiment 2 are 0.90 and 0.94, respectively. The values are lower in new supplemental study (0.3 and 0.6), but these online data in general are noisier. Overall, the Cohen’s *d* metric would suggest the effect is relatively robust, a conclusion further supported by our expanded review of other relevant studies (see below).

The early learning results are, admittedly more problematic. We first want to highlight that we identified an error in how we analyzed Experiment 1 early learning in the initial submission, one that may have contributed to the impression that the effects are weak. We used a randomization process to determine which trials were Rotation trials and which were Probe trials, and then used the same order for all participants. Due to this randomization, the first trial in the first (but not the second) learning block was a Probe trial, and we treated it as such. However, since the rotation had yet to be presented, it should have been treated as a baseline trial. When we corrected for this mistake, the increase in hand angle for this cycle (comprised of four trials) in the second block (i.e., savings) was no longer significant in the cluster analysis (Fig. 1D). We updated the manuscript accordingly (and added this info to the Data analysis section, lines 729-732).

Nonetheless, a significant increase in relearning is still present in the early learning measure using predefined cycles for the Probe trials (Fig. 1G). We don’t have a ready explanation for this but, as noted in the original manuscript, there may be contamination from switching errors (see our response to Comment #2 of Reviewer #2) as participants start each block and settle into the rhythm of switching between development/recall of an aiming strategy (Rotation trials) to actively avoiding re-aiming (Probe trials). We do recognize that the method in Experiment 1 has limitations in terms of isolating implicit learning during the learning blocks. This motivated Experiment 2 (and now also Exp. 2S, see below) since the clamped-feedback method allows implicit adaptation to be measured directly and does not involve re-aiming on any trials. With this design, there was no significant difference in hand angle between blocks during early learning.

Along the same lines, the effect sizes for the "prior evidence for attenuation upon relearning for implicit visuomotor adaptation" appear small. The main worry here is cherry-picking those adaptation studies that have shown an attenuation effect and ignoring those that have shown no effect or a savings effect. A well-designed comprehensive meta-analysis is needed here to guard against a potentially premature conclusion. Such meta-analysis would include studies in which implicit savings have been shown (e.g., Coltman et al., 2019; Yin and Wei, 2020), studies in which no savings have been shown (e.g. Haith et al., 2015; Leow et al., 2020), and other savings studies that are not included (e.g., Klassen, et al. 2005; Orban de Xivry and Lefèvre 2015; Oh and Schweighofer 2019).

We had selected three studies that provided a clean test of the effect of relearning on implicit adaptation. However, inspired by the reviewer’s comment, we embarked on a more comprehensive review. A true meta-analysis is not possible here in the sense that methods designed to isolate implicit learning are relatively new (since ~2014) and thus the number of relevant papers is limited. Nonetheless, we did comb the literature to review all of the papers we could find that seemed to provide a reasonable test (i.e., used a method in which implicit adaptation was studied over at least two learning blocks), noting that in some cases, the experiment was not designed to ask about savings/attenuation (and thus the analysis relevant to this question might not have been performed or discussed). This search led us to identify 13 relevant experiments (from 9 studies). For the studies that did not report the relevant analyses, we obtained the original data from the authors and used these to calculate the effect size for the comparison of implicit adaptation across blocks/sessions. We now present the results of this review in Figure 3, also including some panels to illustrate attenuation effects in different paradigms (Fig. 3B-3D). As can be seen in the figure, the overall pattern is one of attenuation for late learning and aftereffect, usually with at least a medium effect size, and again, a mixed pattern for early learning. We suspect the inconsistency in the early learning results arises in part, from the fact that the level of learning is generally low during this phase, making it less sensitive for revealing changes between blocks. Moreover, any failure to completely wash out the effects of adaptation from the first block will introduce a bias towards savings.

***Figure 3. Prior evidence for attenuation upon relearning for implicit visuomotor adaptation.***

*(****A****) Cohen’s d effect size of the differences in learning measures between the second and first learning blocks for experiments meeting our criterion for inclusion in the review of the literature. Early learning (green circles), Late learning (blue triangles), Aftereffect (purple squares) and Learning rate (dark purple stars). (****B, C****) Overlaid hand angle functions of implicit adaptation to a visuomotor rotation over two learning blocks separated by a washout phase (not shown). Light and dark colors denote blocks 1 and 2 of the experiment, respectively. Shaded margins represent the SEM. In B, implicit adaptation was extracted by subtracting a reported aiming location from movement hand angle on every trial. Adapted with permission from Yin and Wei, 2020. In C, implicit adaptive response to the rotated cursor when the target jumps in a manner that eliminates task error. Adapted with permission from Leow et al., 2020. (****D****) Learning rate during adaptation to a gradually changing visuomotor rotation, tested on two days. Pink markers represent individual participants. Black diagonal dotted line represents the unity line. Bars and black vertical lines (inset) represent mean and SEM, respectively. Adapted with permission from Stark-Inbar et al., 2016.*

We again note that the attenuation effect had been overlooked in most of these studies, or only briefly noted because it was secondary to the focus of the study. We also assume that the effect may have been discounted because of the strong expectation for savings (or no change). One example here that is especially important to highlight is that from Yin and Wei 2020 which claims for a positive effect of implicit adaptation to savings when explicit strategies are also involved in the second learning block, but in fact, also shows unappreciated attenuation when examining the implicit process upon relearning (Fig. 3B). The review does complement the current work in showing that the attenuation phenomenon is present in studies from different laboratories, different experimental setups, and different task designs. In terms of organization, we have moved the review to the Discussion section (a suggestion made by Reviewer 2, lines 307-415).

There are cases where attenuation was not observed, including cases where the relearning data are consistent with savings. All of the cases showing a moderate effect size in the direction of savings used the design of Experiment 1, where participants alternative between Rotation and Probe trials (Morehead et al., 2015, Exp. 4, Vandevoorde and Orban de Xivry, 2019, Exps. 1a and 1b). As discussed above, this method may be problematic for isolating implicit learning due to switching errors. We discuss these exceptions in the revision (lines 395-404).

One last comment on our expanded review is that there are a few studies that the reviewer had suggested, but we excluded because we did not think they provided a real test of the effect of relearning on implicit adaptation.

- Coltman et al. 2019: The claim that the implicit system contributes to savings in a force field adaptation is based on fitting the data to a dual-rate model with the assumption that the slow system solely reflects implicit learning. Not only is this a strong assumption, but our selection criteria required studies in which relearning effects could be obtained from the behavioral results.
- Klassen et al. 2005: The change in behavior for the groups that experienced the gradual perturbations can be considered as implicit (also verified by a post-experiment debriefing). However, the gradual schedule in these groups was only presented in the first session; on the second session, all participants experience an abrupt perturbation. Thus, this study does not assess how the implicit system changes between the sessions.
- Orban de Xivry and Lefèvre 2015: The design of this study is optimized to assess anterograde interference rather than relearning/savings, with the perturbation on the second learning block always in the opposite direction of the initial perturbation.
- Oh and Schweighofer 2019: This study did not use a procedure to dissociate implicit and explicit processes.

Finally, the implicit component of visuo-motor adaptation is thought to be a cerebellar-dependent process. Because the results presented are opposite to previous results showing savings in other cerebellar-dependent processes (such as eye-blink conditioning and saccadic adaptation, as cited), additional solid evidence from well-designed experiments that specifically aim at studying savings in the implicit component, such as experiment 2, are needed.

This comment, along with one from Reviewer 2, motivated us to conduct a new experiment. Given the pandemic, we ran an online version of Experiment 2 with clamped feedback. While online testing means that there will be considerable variability in the experimental setup between individuals, the results were quite similar to that observed in Experiment 2, showing attenuation in the late phase of learning and in the aftereffect block. This study is presented as a supplemental experiment (Experiment 2S, Fig. S2, shown below). When the three studies in the paper are considered in tandem with the expanded review of other relevant studies, we think the evidence is quite compelling that implicit adaptation is attenuated during relearning for visuomotor adaptation.

***Figure S2. Experiment 2S: Replication study using an online crowdsourcing platform.***

*(****A****) Time course of participants’ (N=48) mean hand angle averaged over cycles, showing robust adaptation to the task-irrelevant clamped feedback (onset marked by the vertical lines) in blocks 1 (light green) and 2 (dark green) (Early-Late Stage main effect: F(1,47)=90.8, p<0.001, η_p_^2^=0.66). (****B****) Overlaid hand angle functions for the two blocks, showing attenuation in multiple clusters throughout the learning and aftereffect blocks (p<0.05). While the effect of Block was not significant (F(1,47)=1.09, p=0.302, η_p_^2^=0.02), there was a significant Stage x Block interaction effect (F(1,47)=4.99, p=0.030, η_p_^2^=0.10). (****C-D****) The interaction reflects the finding that there was a significant attenuation in the Late stage (-1.57°±0.74°, t(47)=-2.14, p=0.038, BF10=1.25, d=-0.31), but no difference in the Early stage (0.58°±0.61°, t(47)=0.950, p=0.347, BF10=0.240, d=0.14). (****E****) Similar to the results of Experiments 1 and 2, marked attenuation was observed in the Aftereffect stage (-3.35°±0.81°, t(47)=-4.14, p<0.001, BF10=168, d=-0.60). For panels C-E, the left pair of bars show the mean across participants for each block, and the right bar shows the mean of the within-participant differences Block 2 – Block 1. Black dots indicate individual difference scores. For all figure panels, shaded margins and black vertical lines represent standard error of the mean (SEM).*

The question of whether attenuation in relearning is a general feature of cerebellar-dependent learning is an interesting one, and looking across diverse tasks is an appropriate approach for addressing this question. We believe that the evidence for savings in saccade adaptation is not strong when the experiments include an extended washout period (Kojima et al. 2004), as well as in eye-blink conditioning when the extinction phase involves the repeated presentation of the CS and the US in an unpaired manner (Frey and Butler 1977). Indeed, in one study of fear conditioning, another learning process associated with the cerebellum, the duration of the extinction phase was a key independent variable (Bouton 1986). Here the results show that with a long extinction phase, there is actually attenuation during relearning. This finding is consistent with the interference mechanism we suggest for attenuation. We discuss these examples in the paper (lines 470-491).

Minor

The two sub-groups of experiment 1 are not justified. Indeed, the rotation and target positions of the first sub-group are not ideal, since the "correct answers" are shooting to the cardinal directions. Too much strategy is involved here. Is there a difference in results between the sub-groups?

We opted to include the two subgroups because of this very concern, thinking that certain configurations may be more conducive for finding the optimal re-aiming strategy. As noted by the reviewer, for a diagonal layout (target positions at 45°, 135°, 225°, 315°), offsetting a 45° rotation could be achieved by moving along the cardinal directions (although given the on-going operation of implicit adaptation, this strategy only works for the first few movements). We thought it is important to include this layout since it has been used in many prior studies (e.g. Krakauer et al. 2000, Taylor et al. 2014) but also added the other configuration (target positions at 20°, 110°, 200°, 290°) as a point of comparison. We did not find a difference between the two subgroups on any measure, including the amount of savings observed on the Rotation trials (where the effect is driven by aiming) (Early learning: t(22)=-0.57, p=0.576; Late learning: t(22)=-1.98, p=0.059). We added this information to the manuscript (lines 577-582, 717-720).

Only the results of statistical tests are reported. We need to see means, standard deviations, and effect sizes.  The presumably small effect sizes overall in both experiments are troubling from a reproducibility point of view.

We added this information for all of the reported results. As discussed in detail in our response to the reviewer’s comment above, the effects sizes for attenuation generally fall in the moderate to large range, at least for the late learning and aftereffect data.

The main statistical test used is the uncorrected t-test. Given the large number of tests performed there is a large probability of chance findings. Consider using a correction such as the FDR. In addition, it is unclear whether the normality assumptions have been verified.

In our statistical analyses, there were two primary ways in which we used t-tests. One was part of the *a priori* plan to perform simple effect comparisons for predefined cycles (e.g., Early/Late learning), comparisons that allow us to elucidate the source of an interaction in the ANOVA (which we added to the results following Comment #3 of Reviewer #1). For these, we did not correct for multiple comparisons, followed the convention for our planned contrasts. Using the Lilliefors test, we verified that the normality assumption was met in all of these t-tests. We have added this information in the revision (lines 773-774).

The second approach is the cluster-based permutation analysis. This analysis is designed to address the problem that pre-defined cycles are somewhat arbitrary in nature and ignore a good bit of the data. This test seems well-suited for sensorimotor learning studies given the high autocorrelation between sequential data point observed in these studies and incorporates a procedure that corrects for multiple comparisons. The cluster-based permutation approach is a non-parametric test that does not require that the data for each cycle be normally distributed (Maris and Oostenveld 2007).

I like in principle the permutation approach, but it also seems to pick up noise. See for instance the small cluster of significant difference around 25 trials in Figure 1D. Showing the p values instead of the p<0.05 threshold, as well as the effect size, may help here.

We thank the reviewer for this observation and suggestion. We now use a more conservative approach where all the candidate clusters found in the original data are evaluated with respect to the permutation distribution of the maximum t-sum values (Maris and Oostenveld 2007). This solves the multiple comparison problem and reduces the sensitivity of the test to small clusters.

This alternative approach for the permutation test did produce a few, relatively minor changes in comparison to what we reported in the original version. The small clusters in the Probe trials of Experiment 1 did not survive the more conservative analysis, an effect that had been weak (as noted by the reviewers). In contrast, pronounced clusters remain significant in the aftereffect block of all three experiments (Exp 1, 2, and 2S), as well as in the learning phase with clamped-feedback in Experiments 2 and 2S.

Reporting exact p-values and effect sizes is not conventional with the permutation test. This procedure relies on randomization of the data; as such, each run of the test yields different values (within a small range). For this reason, we report clusters based on the predetermined 0.05 threshold.

Line 73: Neither I, nor the authors in line 180, agree with this statement

We revised the statement (line 74-76):

**“These probe trials are designed to assay the state of implicit adaptation (Huberdeau et al., 2019; Morehead et al., 2015; Vandevoorde and Orban de Xivry, 2019).”**
