# Supplementary material for: Reexposure to a sensorimotor perturbation produces opposite effects on explicit and implicit learning processes
Source: PLoS Biol. 2021 Mar 5;19(3):e3001147. doi: 10.1371/journal.pbio.3001147 (PMC7968744; doi:10.1371/journal.pbio.3001147)
Supplement: Attachment — Submitted filename: Avraham_ResponseToEditor.docx [file pbio.3001147.s006.docx]

RE: Manuscript Number PBIOLOGY-D-20-02151R3

**Editor:**

ETHICS STATEMENT:

-- Please include in your manuscript the ID number of your protocol approved by the Institutional Review Board at the University of California, Berkeley.

We have added the protocol ID number to the manuscript.

-- Please indicate in your manuscript if your approved protocols adhered to the principles expressed in the Declaration of Helsinki or to any other national or international ethical guidelines.

We have updated the statement to indicate that the protocols adhered to the principles expressed in the Declaration of Helsinki.

------------------------------------------------------------------------
DATA POLICY:

You may be aware of the PLOS Data Policy, which requires that all data be made available without restriction: <http://journals.plos.org/plosbiology/s/data-availability>. For more information, please also see this editorial: <http://dx.doi.org/10.1371/journal.pbio.1001797>

I note that you have provided your data in the GitHub repository:

<https://github.com/guyavr/ReexposureSensorimotorPerturbationProducesOppositeEffectsExplicitImplicit>”

Please update the README file to include detailed information on how your data were analyzed to generate the plots shown in figures 1B-I, 2CJ, 3A-D, S1, and S2A-E.

Alternatively (or in addition) you can provide a spread sheet with all individual quantitative observations used to generate the plots listed above. For an example see here: <http://www.plosbiology.org/article/info%3Adoi%2F10.1371%2Fjournal.pbio.1001908#s5>

The numerical data provided should include all replicates AND the way in which the plotted mean and errors were derived (it should not present only the mean/average values).

We now provide a spreadsheet with the individual data and the summary statistics used to generate all the plots. We also updated the README in the GitHub repository which includes the raw data from all of the experiments, along with a detailed description of the variables.

Please also ensure that each figure legend in your manuscript includes information on where the underlying data can be found and that your supplemental data file/s has/have a legend.

This information has been added to the figure legends.

Please ensure that your Data Statement in the submission system accurately describes where your data can be found.

We have confirmed that the Data Statement is accurate and describes where the data can be found.
